# Rotational Changes in the Distal Tibial Fragment Relative to the Proximal Tibial Fragment at the Osteotomy Site after Open-Wedge High-Tibial Osteotomy

**DOI:** 10.1155/2021/6357109

**Published:** 2021-02-01

**Authors:** Takahiro Sasaki, Yasushi Akamatsu, Hideo Kobayashi, Shota Mitsuhashi, Shuntaro Nejima, Ken Kumagai, Tomoyuki Saito, Yutaka Inaba

**Affiliations:** Department of Orthopaedic Surgery, Yokohama City University School of Medicine, 3-9 Fukuura, Kanazawa-ku, Yokohama, Japan

## Abstract

The present study is aimed at assessing the changes in tibial rotation at the osteotomy site after an open-wedge, high-tibial osteotomy (OWHTO) and analysing the factors that affect rotational changes in the distal tibial fragment relative to the proximal tibial fragment at the same site. This study involved 53 patients (60 knees; 16 males and 37 females) with medial osteoarthritis (OA) who underwent OWHTO and preoperative and 3-month postoperative computed tomography (CT) scans. Rotational angles of the distal tibia were measured using Stryker OrthoMap 3D by comparing preoperative and postoperative CTs. The mean rotational angle yielded an external rotation of 2.9° ± 4.8°. There were 17 knees with internal rotations, 37 knees with external rotations, and one knee with no rotation. The rotational angle significantly correlated with the resultant change in the femorotibial angle (correction angle) and the angle between the ascending and transverse osteotomy lines on the anterior osteotomised surface on which a flange was formed with the distal tibial osteotomised surface (flange angle). The flange angle affected the rotation, but it may have been affected by our surgical technique. The rotational angle did not significantly correlate with the change in the angle of the posterior tibial slope or body mass index. There were significant correlations between the rotational angle and correction angle (*r* = 0.42, *p* < 0.05). Additionally, the rotational angle correlated with the flange angle (*r* = −0.41, *p* < 0.05).

## 1. Introduction

Open-wedge high-tibial osteotomy (OWHTO) is an established treatment for medial knee osteoarthritis (OA) [1–5]. Its most notable advantages include the precision of intraoperative angular correction, absence of the risk of peroneal nerve palsy in fibular osteotomy, absence of leg shortening, and the preservation of the knee joint. This procedure shifts the loading distribution from the affected medial compartment to the healthy lateral compartment of the knee, and thus leads to a decrease in the symptoms related to the medial compartment of the knee. Accurate preoperative planning and surgical techniques are required to achieve successful outcomes [6]. The focus was based on coronal plane correction. Thus, the sagittal or coronal planes were not considered [7]. However, surgeons considered the tibia a three-dimensional structure that can be visualised along the coronal, sagittal, and axial planes and that alignment changes occur. Unintentional secondary changes at the osteotomy site, such as changes in the posterior tibial slope [8, 9], joint line convergence angle [10, 11], femorotibial subluxation [12], and rotational changes in the distal tibial fragment relative to the proximal tibial fragment at the osteotomy site are possible consequences of OWHTO. Rotational changes in the distal tibial fragment relative to the proximal tibial fragment at the osteotomy site have not been studied in depth [13]. A few studies have reported the rotation of the distal tibial fragment relative to the proximal tibial fragment after OWHTO [13–16]. Hinterwimmer et al. have reported that the mean internal rotation of the distal tibia was 4.4 ± 2.8° [14]. Kendoff et al. found an external rotation of the distal tibia of 2.7 ± 6.3° after OWHTO [16]. In 1999, Magyar et al. [17] first reported axial tibial rotations following closed-wedge, high-tibial osteotomy by callus distraction in 33 consecutive patients. The opening gap affected the tibial rotation at the osteotomy site after OWHTO [15]. These studies reported that the opening width, the opening angle, and posterior tibial slope were related to rotational changes in the distal tibial fragment relative to the proximal tibial fragment at the osteotomy site. However, no prior studies reported the angles between the transverse and ascending osteotomy lines on the sagittal plane. We predicted that the angles between the transverse and ascending osteotomy lines on the sagittal plane were related. This study aimed to investigate the rotation of the distal tibial fragment relative to the proximal tibial fragment after OWHTO. We hypothesised that the rotational change in the distal tibial fragment relative to the proximal tibial fragment at the osteotomy site was related to the angle between the transverse and ascending osteotomy lines on the sagittal view, and the changes in the coronal alignment after OWHTO.

## 2. Materials and Methods

This study was approved by the institutional review board of Yokohama City University (B150108021), and informed consent was obtained from each patient. A cross-sectional study at our institution included 60 knees of 53 patients (37 females and 16 males) with medial OA who underwent OWHTO between February 2014 and March 2016 at our department. All participants agreed with the study protocol, including the CT evaluation. Initially, 65 patients (70 knees) agreed to participate in the study. However, 12 patients (10 knees) did not undergo preoperative or postoperative CT scans. Consequently, these patients were excluded from the study. Thus, the present study cohort comprised 53 patients (60 knees). An experienced orthopaedic surgeon performed all the operations. Only knees subjected to preoperative and postoperative CT scans were included in the study. The eligibility criteria for OWHTO were spontaneous osteonecrosis of the knee of the medial femoral condyle, medial compartment OA, Ahlbäck grades [18] 1 or 2, femorotibial angle (FTA) of ≤185°, flexion contracture of ≤15°, lack of damage to the anterior cruciate ligament (ACL) and posterior cruciate ligament, and no age restrictions [19, 20].

### 2.1. Surgical Technique

We used preoperative planning to align the FTA at 170° and to obtain a mechanical axis of the lower extremity that passed through the point at 62% of the tibial plateau from the medial edge [21, 22]. Additionally, we used a preoperative standing anteroposterior full-length lower limb radiograph. Arthroscopic examinations were used to assess the status of the articular cartilage, menisci, and cruciate ligaments. If necessary, meniscal procedures, such as meniscectomy, were performed. Using an intraoperative navigation system (OrthoPilot®, Aesculap, Germany), a 4–5 cm vertical skin incision was made medial to the patellar ligament and tibial tubercle. The medial collateral ligament was released, the pes anserinus was conserved, and osteotomy was performed 35 mm distal to the medial proximal joint surface, ending at the safe zone of the lateral side [23]. The osteotomy line in the sagittal plane was 15 mm posterior from the patella tubercle surface, and the length of the sagittal plane osteotomy was approximately 15–20 mm. Two Kirschner wires were inserted along the planned plane under fluoroscopic control, and the osteotomy was performed with a bone saw that operated distally to the Kirschner wires to avoid proximal migration of osteotomy into the joint. After the osteotomy, the osteotomy site was opened using a bone spreader at a predetermined distance. During sectioning, care was exercised with visual inspection such that the proximal and distal tibia fragments were in contact at the flange. With the use of an intraoperative navigation system, alignment was corrected and an appropriately sized *β*-TCP (OSferion60®, Olympus, Japan) was fitted to the opening site [22]. Internal fixation was then performed with a locking plate (TomoFix, DePuy Synthes, Bettlach, Switzerland) and screws. We simultaneously monitored the TPS to prevent TPS changes in the sagittal plane.

### 2.2. Postoperative Rehabilitation

All patients received postoperative venous impulse foot pumping. Quadricep exercises and knee and ankle joint motion exercises were initiated on the first day after surgery and were supervised by a physical therapist. Walking exercises with full weight bearing began one week after surgery.

### 2.3. X-Ray Measurements

**Figure 1:**
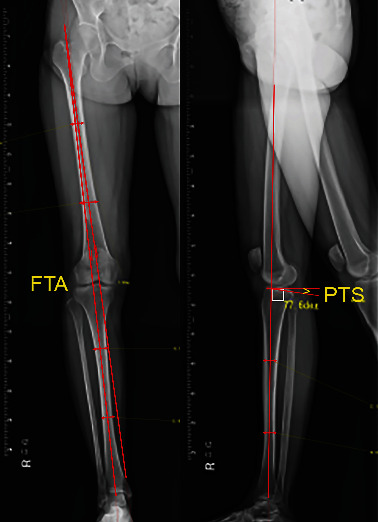
Measurement of the femorotibial angle and the posterior tibial slope angle. The femorotibial angle (FTA) was defined as the lateral angle between the femoral anatomical shaft axis and the tibial anatomical shaft axis on the long leg radiograph at the coronal plane. The posterior tibial slope (PTS) was defined as the posterior angle between the tibial anatomical shaft axis and tibial plateau on the long leg radiograph at the sagittal plane.

Preoperative and three-month postoperative X-rays of the lower limb were obtained during single-leg weight bearing. X-ray measurements were obtained using a Fuji computed radiography system. We measured the correlation between the rotational angle and resultant changes in the FTA (correction angle) and the posterior tibial slope (PTS) changes using the Fujifilm OP-A software (Fujifilm Co. Ltd., Tokyo, Japan) (Figure 1).

### 2.4. CT Measurements

**Figure 2:**
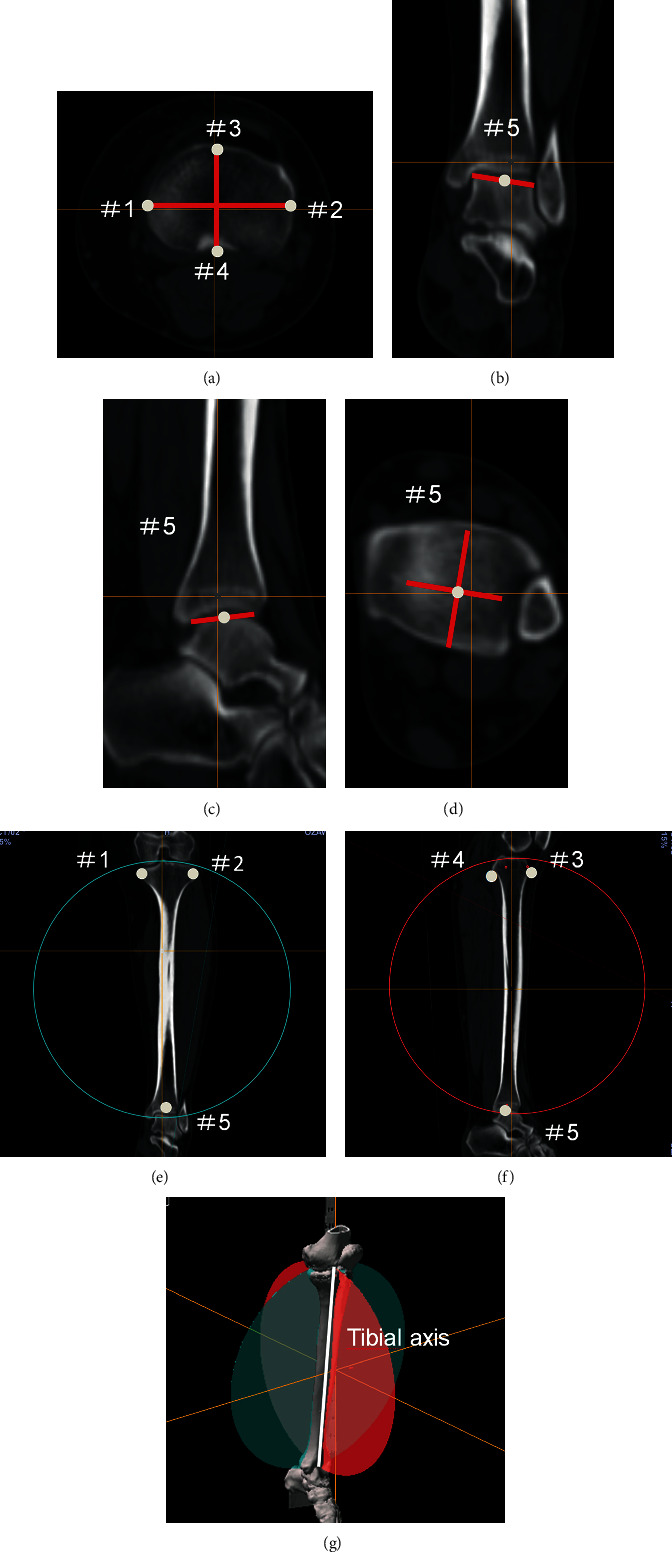
Determination of the tibial axis. On the tibial image of the axial plane, we determined the line that connects the middle of the posterior cruciate ligament and the medial edge of the patellar tendon attachment to be the Akagi line. As noted, #1 and #2 are the points where the vertical line to the Akagi line crosses the medial and lateral cortical bone. In addition, points #3 and #4 are the points where the Akagi line crosses the anterior and posterior cortical bone (a). Determining the ankle centre on the axial plane required identifying the talar dome weight-bearing area of the distal tibial articular surface. Firstly, we drew a long axis on the long axis of the talar dome on the image of the distal tibial joint (b). On the sagittal plane, we drew the long axis on the talar dome (c and d) with the point of the intersection being #5 (the ankle centre). The reference coronal plane is shown (the green plane passing through #1, #2, and #5) (e). The reference sagittal plane is shown (the red plane passes through #3, #4, and #5) (f). These two planes are defined as the AP planes. The intersecting line of the two planes is defined as the tibial axis (g).

**Figure 3:**
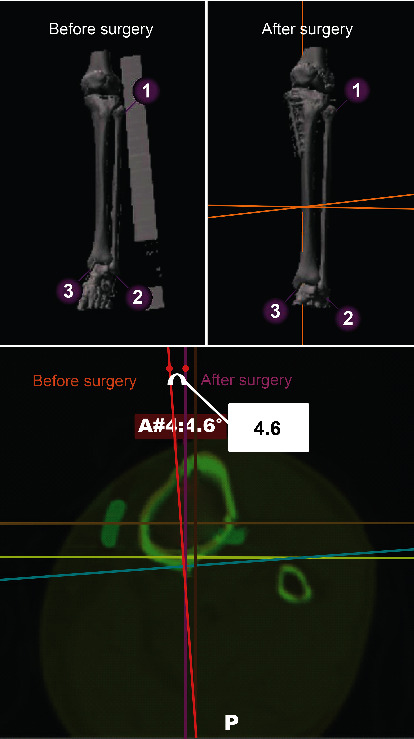
Positioning of matched images. (1) proximal fibula, (2) distal fibula, and (3) medial malleolus before and after the operation are shown. The rotational angle in this case was 4.6° (internal rotation).

**Figure 4:**
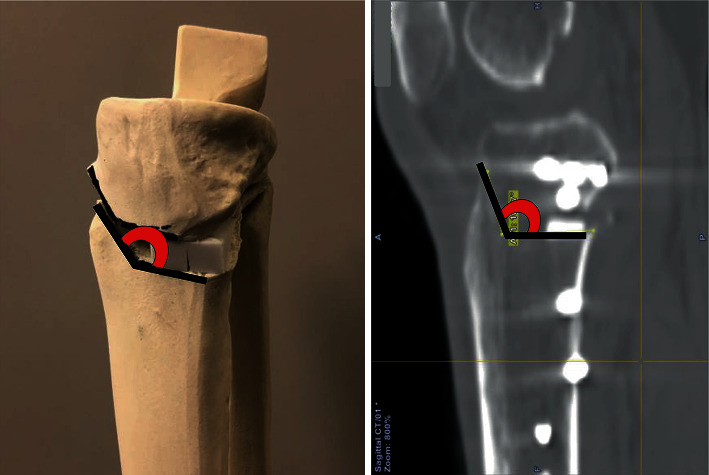
Measurement of the flange angle. We measured the angle between the ascending and transverse osteotomy lines on the anterior osteotomised surface where the flange was formed with the distal tibial osteotomised fragment (this study defined it so that sagittal plane on the CT could pass through the most anterior point of the tibial tuberosity).

Preoperative and postoperative (at three months) CT scans were acquired (Siemens SOMATOM Definition AS+, with a slice thickness of 5 mm). The images were superimposed with the OrthoMap 3D software (Stryker, Kalamazoo, MI, USA). A reference plane was used to determine the tibial axis. The Akagi line [24] was determined from the tibia image (Figure 2(a)). After determining the ankle centre according to the study by Yoshida et al. [25] (Figures 2(b)–2(d)), we set the talar dome weight-bearing area. On the tibial plateau, we determined the intersection point of the coronal axis and cortical bone and the intersection point of the sagittal axis and cortical bone. We then determined the reference plane that passed through these two points and the ankle centre. The point of intersection of the coronal and sagittal plane lines was defined as the tibial axis (Figures 2(e)–2(g)). We superimposed the preoperative and three-month postoperative tibial axes and marked and compared three points (proximal fibula, distal fibula, and medial malleolus) on each tibia (Figure 3). By superimposing the distal tibia images, we measured the rotation between the proximal and distal tibial fragment. The rotational angle was determined to be the distal tibial fragment relative to the proximal tibial fragment at the osteotomy site on the axial plane, and was expressed in terms of either the internal or external rotations. The intersecting angle of the preoperative and postoperative sagittal planes (the Akagi line projected on the plane vertical to the tibial axis) was defined as the rotational angle (Figure 3). This study defined it so that sagittal plane on the CT can pass through the most anterior point of the tibial tuberosity. Accordingly, the angle between the transverse and ascending osteotomy lines on the sagittal plane (flange angle) was measured (Figure 4).

### 2.5. Statistical Analysis

SPSS for Windows (version 20, IBM, Armonk, NY, USA) was used for all statistical analyses. *p* values less than 0.05 were considered significant. A t-test was used to examine whether there was a tibial rotation change before and after OWHTO. A power analysis was performed on correlations (*r* = 0.3, significance level = 0.05, sample size = 60) using G∗Power (version 3.1.9.2, Heinrich-Heine-Universität, Düsseldorf, Germany). A post-hoc power analysis resulted in a power of 0.81. Data were expressed as means ± standard deviations. The Pearson regression coefficient was used to evaluate the association of the rotational angle with body mass index, correction angle, correction loss angle, TPS change, and flange angle. The intra- and interobserver reliabilities of each measurement were assessed by determining the intraclass correlation coefficient (ICC). The intra- and interobserver ICC values were 0.68 and 0.58 for the rotational angle, 0.81 and 0.72 for the correction angle, 0.87 and 0.69 for the PTS angle, and 0.69 and 0.61 for the flange angle, respectively. The scoring system by Landis et al. was used to analyse the results [3].

## 3. Results

**Figure 5:**
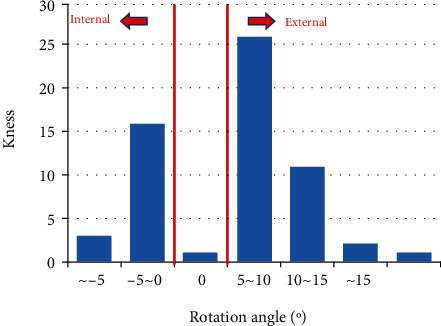
We found both external (*n* = 40) and internal (*n* = 19) rotations in the knees and no rotations in one knee. The “+” sign denotes external rotation.

Upon examination of the rotational directions of the distal tibial fragment relative to the proximal tibial fragment, we found the external rotations in 40 knees, internal rotations in 19 knees, and no rotations in one knee. The mean rotational angle was an external rotational angle of 2.6 ± 4.7° (Figure 5). A significant rotational change was documented regarding the rotation in the distal tibial fragment relative to the proximal tibial fragment at the osteotomy site (*p* < 0.05). The patients' demographic information is summarised in Tables 1 and 2.

**Figure 6:**
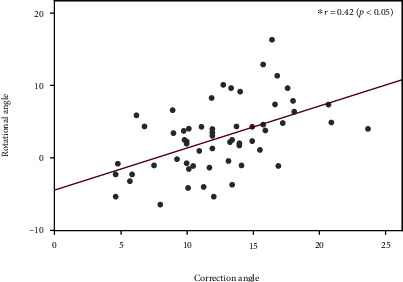
Factors affecting the rotational angle. There is a significant correlation between the rotational and correction angles (*r* = 0.42, *p* < 0.05).

**Figure 7:**
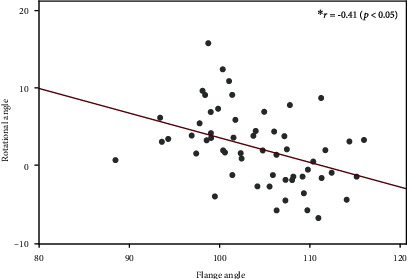
Factors affecting the rotational angle (flange angle). There was a significant correlation between the rotational and the flange angles (*r* = −0.41, *p* < 0.05).

There were significant correlations between the rotational angle and correction angle (*r* = 0.42, *p* < 0.05) (Figure 6). Additionally, the rotational angle correlated with the flange angle (*r* = −0.41, *p* < 0.05) (Figure 7).

## 4. Discussion

The most important finding of this study was that the flange and the correction angles affected tibial rotation at the osteotomy site. A few studies have reported the rotation of the distal tibial fragment relative to the proximal tibial fragment after OWHTO [13–16]. Hinterwimmer et al. in their study adjusted the TPS with two parallel K-wires that were inserted into the proximal and distal tibial fragment [14]. They reported a mean internal rotation of the distal tibia of 4.4 ± 2.8°. These data included low-tibial osteotomy for a patient with a patellofemoral disorder and OWHTO combined with ACL reconstruction for a patient with knee OA and ACL injuries. Additionally, they independently measured the rotation of the two K-wires with a sterile goniometer. However, this was different from the three-dimensional (3D) evaluation with CT employed in our study. This methodological variation may explain the observed differences in the rotational change in the distal tibial fragment relative to the proximal tibial fragment at the osteotomy site between their study and ours. Kendoff et al. in their study performed navigated HTOs on tibial specimens and recorded preoperative and postoperative alignments with a conventional navigation system (BrainLAB, Feldkirchen, Germany). They compared the planes on preoperative and postoperative 3D CTs and found an external rotation of 2.7 ± 6.3° after OWHTO [16]. However, they used fresh-frozen cadavers and a different type of plate.

Jang et al. in their study reported that the rotational angle significantly correlated with the correction angle, but the rotational angle did not correlate with the PTS change [15]. They controlled for TPS in the sagittal view using fluoroscopy. However, our study controlled for TPS with an intraoperative navigation system. We superimposed preoperative and postoperative 3D CT images and measured the rotation between the proximal and distal tibial fragment in the axial view. In the study by Jang et al. and ours, there was no correlation between the rotation angle and PTS.

**Figure 8:**
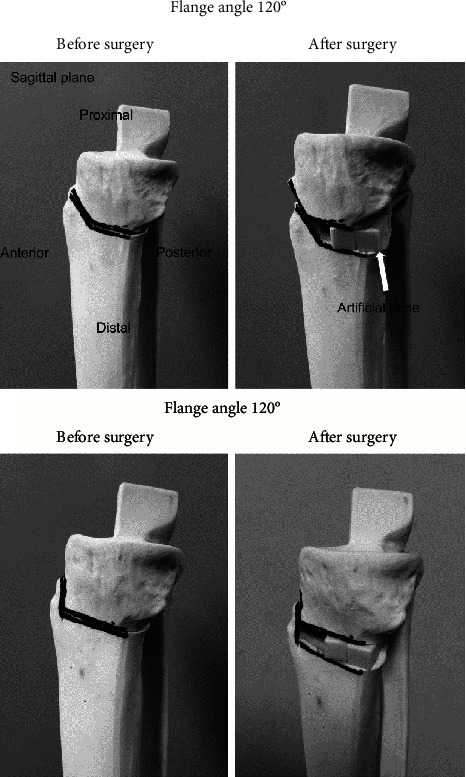
Rotational change per flange angle. A right tibial model is used to show the rotational change per flange angle. The model compared the flange angles of 100° and 120°.

This study indicated that the tibial rotation change at the osteotomy site after OWHTO correlated with the correction angle. Therefore, it is important not to increase the flange angle because higher flange angles result in internal rotational angles (Figures 7 and 8). Additionally, higher correction angles resulted in higher external rotation angles. In other words, when the correction angle is large, the rotational change can be reduced by increasing the flange angle. Theoretically, the angle of the internal rotation of the distal tibia becomes smaller as the flange angle decreases, and the rotation should not occur at 90° (Figure 8). However, in the present study, external rotation occurred as the flange angle decreased, and was observed at a flange angle of 90° (Figure 7). Although no rotation at a flange angle of 90° actually occurred, the flange angle of 90° was clinically challenging to achieve. Our results indicated that angles in the range of 100–110° are appropriate to achieve small rotational angles. Therefore, rotational and flange angles correlated significantly, but they might have been affected by our surgical technique. This is because the rotation angle is not easily affected by the flange angle unless a contact force is applied.

Regarding the rotational angle after OWHTO at the osteotomy site, there are reports of internal [14, 15] and external rotations [16], despite the fact that in this study we only observed external rotations. This showed that this study has an influence on the rotational change in the distal tibial fragment relative to the proximal tibial fragment at the osteotomy site owing to the change of the flange angle. There are some possible factors that affect rotational changes in the distal tibial fragment relative to the proximal tibial fragment at the osteotomy site. Once the osteotomy site was spread open, tension could develop around the intact fibular or tibiofibular joints [26]. Unless the fibular or tibiofibular joints were disrupted, rotational changes in the distal tibial fragment relative to the proximal tibial fragment at the osteotomy site would inevitably develop to some extent to relieve that tension by opening the osteotomy site gap. The direction of the rotation of the distal tibia could be determined by the tension in the soft tissue envelope of the proximal tibia, such as the hamstring tendon, medial collateral ligament (MCL), or joint capsule. The hamstring tendon acted as an internal rotator of the tibia and the MCL to prevent tibial rotation. Therefore, differences in the management of these key medial structures of the proximal tibia during surgery could affect distal tibial rotation following OWHTO [27]. The reason for the internal rotations may be attributed to the lateral flange osteotomy gap opening. However, further consideration is needed as the hinges, the centres of rotation, differ among patients, and the proximal tibiofibular ligament may also exert an effect. The cause of the external tibial rotation at the osteotomy site is attributed to the fact that the internal osteotomy gap between the transverse and ascending osteotomy lines on the sagittal plane may open subject to the influence of the inserted screw during the plate fixation. Higher soft tissue tension on the lateral side of flange as well as the lateral and posterior positions of the proximal tibiofibular ligament may affect external rotations [16]. More studies are needed to be conducted because of the possible transverse cut along the tibial axial plane.

Our study is associated with some limitations. First, our results failed to demonstrate an association between the tibial rotational changes and clinical endpoints. Therefore, we could not discuss the clinical impact of our results or state how large rotational changes relate to clinical outcomes. Second, we only evaluated OWHTO. To clarify rotational changes more accurately, studies that compare OWHTO with closed-wedge HTO should be performed. Although this is a different procedure, it may help us clarify the involvement of soft tissues and ligaments surrounding the osteotomy site. Third, in this study, we examined the relationships between rotational and correction angles and the flange angle. However, we were unable to investigate related factors that may affect the rotational angle.

## 5. Conclusions

The external rotational angle was 2.9° ± 4.8° after OWHTO. The rotational angle significantly correlated with the correction and the flange angles. However, the rotation angle may be affected by the flange angle in addition to our surgical technique of bringing the bones in contact with each other at the flange during the operation.

## Figures and Tables

**Table 1:** Preoperative data.

	Mean (range) *n* = 60
Female/male	42/18
Age (year)	64.4 ± 8.8 (42–80)
BMI (kg/m^2^)	25.9 ± 4.1 (17.3–35.3)
Femorotibial angle (°)	180.9 ± 3.2 (173.4–184.7)
Weight-bearing line ratio (%)	17.4 ± 12.2 (0.3–49.2)
Posterior tibial slope (°)	10.3 ± 3.2 (3.6–17.2)

Data are presented as means ± standard deviation with the range in parentheses. BMI: body mass index.

**Table 2:** Postoperative data.

Maximal externally rotational angle (°)	16.1
Maximal internally rotational angle (°)	6.6
Femorotibial angle (°)	168.5 ± 2.8 (162.2–173.9)
Weight-bearing line ratio (%)	68.5 ± 10.6 (46.9–86.3)
Correction angle (°)	12.2 ± 3.8 (5.0–23.8)
Tibial posterior slope (°)	10.8 ± 3.9 (0–17.1)
Change in posterior tibial slope (°)	3.9 ± 0.4 (−9.1–9.3)
Flange angle (°)	104.6 ± 6.2 (88.8–117.2)

Data are presented as means ± standard deviation with the range in parentheses.

## Data Availability

The data used to support the findings of this study are included within the article.
